# Supplementary material for: Structural and Functional Glycosylation of the Abdala COVID-19 Vaccine
Source: Glycobiology. 2025 Jan 12;35(3):cwaf001. doi: 10.1093/glycob/cwaf001 (PMC11758712; doi:10.1093/glycob/cwaf001)
Supplement: Cover_Letter_Revision_Struwe_cwaf001 [file cover_letter_revision_struwe_cwaf001.pdf]

DEPARTMENT OF BIOCHEMISTRY

Weston Struwe PhD
Associate Professor of Biochemistry
Tutorial Fellow at Worcester College
UKRI Future Leaders Fellow
Kavli Institute for NanoScience Discovery
Dorothy Crowfoot Hodgkin Building, Oxford, OX1 3TA, UK
Tel: +44(0)1865 285445; Email: weston.struwe@bioch.ox.ac.uk

UNIVERSITY OF OXFORD

2nd January 2025

Dear Prof Imberty,

We thank you for considering our manuscript "*Structural and Functional Significance of Abdala COVID-19 Vaccine Glycosylation*" for publication. We are grateful to the reviewers for their constructive comments, and we feel that our amendments to the manuscript add clarification where needed. All changes to the manuscript as suggested by both Reviewers are addressed point-by-point below.

We hope you will now find our manuscript suitable for publication

Yours sincerely,

Weston Struwe

Reviewer: 1

Comments to the Author

In this manuscript the authors present the results of a thorough characterisation by mass spectrometry (MS) and hydrogen-deuterium exchange (HDX) methods of the N- and O-glycosylation of the RBD-based vaccine construct ‘Abdala’ expressed in P. pastoris (RBD-Abd). The authors identify significant differences in the type of N-glycosylation relative to the RBDHEK, as well as the presence of O-mannosylation, although with relatively low occupancy. While those dramatic differences are expected, their effects on the activity of the biologic are really interesting and of great consequence.

The authors measured the differences in binding affinity of RBD-Abd and RBD-HEK to ACE2 and to an RBM-targeting monoclonal antibody under investigation. They found that the large differences in N-glycosylation affect binding significantly, reducing the overall affinity to both ACE2 and to the antibody. The authors suggest the lower affinity of RDB-Abd can be reconciled with different dynamics of the high-mannose, phosphorylated N-glycans relative to the complex, sialylated forms found in RBD-HEK, which may affect the structure of the RBD and allosterically control the conformation of the distal RBM, which interacts with ACE2 and the antibody. This is in agreement with recently published work cited therein.

I find these results both exciting and impactful as they indicate the key role of N-glycosylation in biologic efficacy and more specifically in this case, vaccine immunogenicity, demanding for a careful choice of cell-line development. For these reasons I strongly support the publication of the manuscript with the (very) minor suggestions below.

As a note, I am unable to comment on the technical details of the research methods in this work as I am not an expert in MS or in HDX.

> *We thank the reviewer for their positive view of our manuscript.*

Minor points

Page 3 line 24. The authors indicate (correctly) that the RBD opening is controlled by two N-glycosylation sites. These are N343 (RBD) and N234 (N-ter), with a minor role of N165 (N-ter). The two references included in the manuscript examine the functional roles of N343, while a (missing) reference: Casalino et al. https://doi.org/10.1021/acscentsci.0c01056 examines the role of both N234 and N165. If the authors meant to include N234 within the two N-glycosylation sites they mentioned in the text, then this reference should be included.

> *We appreciate the reviewer spotting this missing reference. We have now modified the introduction to include this information regarding the three glycans known to regulate RBD dynamics.*

Page 8 line 4. The glycan indicated as “Man5GlcNAc4Fuc1NeuAc2” should probably be indicated with Hex5 instead of Man5, or with Man3Gal2.

> *This has now been changed.*

Reviewer: 2

Comments to the Author

Comment 1 -

Page 3, line 21: The sentence "Spikes are heavily glycosylated, with 66 N-glycans per S trimer with each RBD domain containing 2 N-glycans that modulate protein structural states through “up” and “down” RBD dynamics (Ives et al. 2024; Sztain et al. 2021)." could be rephrased for better clarity.

You mentioned that there are 66 N-glycans per S trimer. I may have missed something. Could you please elaborate on this? Do you mean a total of 66 different N-glycan types across all glycosylation sites, or is this number specific to the N343 site, as the referenced study focused on? Please provide relevant citations to support your claim.

We apologise for the confusion regarding this sentence. This has now been reworded (also in line with comments from reviewer 1) to, “Spikes are heavily glycosylated, with 22 N-glycosylation sites per monomer, equating to 66 N-glycans per S trimer with each RBD domain containing 2 N-glycans, N331 and N343.Glycans at positions N343 (RBD), N234 (NTD) and N165 (NTD) have been shown to modulate protein structural states through “up” and “down” RBD dynamics (Casalino et al. 2020; Ives et al. 2024; Sztain et al. 2021).”

To clarify, the SARS-CoV-2 spike is a trimer of S1 and S2 heterodimers which has 22 N-glycosylation sites (13 on S1 and 9 on S2). Therefore, each trimer has 66 glycans, all of which with varying heterogeneity, as others and ourselves have previously characterised (https://doi.org/10.1126/science.abb9983, https://doi.org/10.1021/acscentsci.1c00058).

Comment 2 -

Page 8, line 53: The peptide at site N331, which resides within the Receptor Binding Domain (RBD) and could potentially influence receptor binding properties, was not detected in the Abdala study, either in its glycosylated or non-glycosylated form. Given that the RBD-Hek construct provides a peptide for this site, it's puzzling why it wasn't identified. Have you experimented with different enzyme combinations for digestion to potentially improve peptide detection?

We tried several proteases to access this site from Abdala, including trypsin, chymotrypsin, alpha-lytic protease, ProAlanase and GluC. However, we believe this site to be occupied based on the mass differences observed when comparing both Abdala and RBD-HEK by SDS-PAGE and mass photometry (see ***Fig 1)****. Furthermore, PNGaseF treatment seemingly resulted in full deglycosylation based on the mass shift seen by SDS-PAGE with both RBD-HEK and Abdala migrating at the same molecular weight post-treatment. Therefore, our analyses includes glycans at this site.*

Other comments -

Page 1, Line 14: Please specify the country in the affiliation.

This has been corrected

Page 1, Line 35: Remove the extra "T" at the end of the line.

This has been corrected

Page 7, Line 17: Please reference a figure or table within the manuscript that illustrates the fragment ions with m/z 259 and 241.

*We have now added an extra supplemental figure highlighting the low mass fragment ions with m/z 259 and 241 (**Supp Fig. S3B**).*

Page 8, Line 3: Were the N-glycan structures confirmed at the MS/MS level for core-fucosylation?

We did not perform a full glycan analysis (i.e. MS/MS) for the HEK-derived RBD as this was not the focus of this report. As such, we cannot exclude the possibility of outer-arm fucosylation for these structures and therefore have removed "core" from these descriptors.

Page 8, Line 14: Please provide a reference for this statement.

The reference Limonta-Fernández et al. 2022 has been added.

Page 14, Line 14: Please elaborate on the rationale for using three lots of the Abdala RBD.

We have added the following statement, "Three production lots of Abdala RBD were assessed in this manuscript to compare consistency in produced protein product,".

Page 14, Line 40: Please specify the full name of the reagent "MeCN: water".

This has been corrected.

Supplementary Table S1: Please clarify whether the HPLC peak area represents absolute values or relative % abundance.

These are absolute values and a comment has been added to the table legend, "HPLC peak areas expressed as absolute values for the 9 major N-glycans from three production batches of Abdala.".
